# A high aminoglycoside regimen associated with renal replacement therapy for the treatment of multi-drug resistant pathogens

**DOI:** 10.1186/2197-425X-3-S1-A404

**Published:** 2015-10-01

**Authors:** A Brasseur, M Hites, S Roisin, F Cotton, J-L Vincent, D De Backer, F Jacobs, FS Taccone

**Affiliations:** ULB, Intensive Care, Brussels, Belgium; ULB, Infectious Diseases, Brussels, Belgium; ULB, Clinical Microbiology, Brussels, Belgium; ULB, Clinical Biochemistry, Brussels, Belgium

## Introduction

Infections caused by multi-drug resistant (MDR) Gram-negative (GN) organisms in critically ill patients are a therapeutic challenge. Few therapeutical options are available.

## Objectives

The administration of high-dose aminoglycoside (HDA) therapy coupled with high-flow continuous veno-venous hemodiafiltration (CVVHDF) could allow required high drug peaks to be achieved with reduced toxicity.

## Methods

All adult patients present on the intensive care unit (ICU) between October 2009 and July 2014 who had MDR-GN sepsis were considered for HDA and high-flow (>45 mL/kg/h) CVVHDF when an isolated pathogen was at least partially sensitive to aminoglycosides and the patient's condition was not improving with conventional therapy. Optimal antibacterial activity was defined as a peak concentration of at least 8 times the minimal inhibitory concentration.

## Results

Fifteen patients infected with MDR-GN pathogens (11 with Pseudomonas aeruginosa; 10 with abdominal and 5 with respiratory infections) were treated with amikacin (n = 11), gentamicin (n = 3) or tobramycin (n = 1) and high-flow CVVHDF. A favorable clinical response was observed in 8 (53%) patients, including 3 in whom microbial eradication was obtained. Six patients were discharged alive from the ICU, and five from the hospital. No renal toxicity was observed.

## Conclusions

In this cohort of septic patients with MDR-GN infections, HDA combined with high-flow CVVHDF represented a valuable therapeutic option. The effectiveness of this approach should be further evaluated in larger studies.

